# Disasters and the education system: Cyclone Idai and schooling disruption in eastern Chimanimani, Zimbabwe

**DOI:** 10.4102/jamba.v15i1.1349

**Published:** 2023-08-10

**Authors:** Happwell Musarandega, Wonder Masocha

**Affiliations:** 1 School of Geoscience, Disaster and Development, Faculty of Science and Engineering, Bindura University of Science Education, Bindura, Zimbabwe; 2 Department of Geography, Marymount Teachers’ College, Mutare, Zimbabwe

**Keywords:** Cyclone Idai, disaster resilience, education system, stakeholders, sustainable development

## Abstract

This article examined how the education system was impacted by the Cyclone ldai disaster in eastern Chimanimani District in 2019. In addition, this article discoursed the enrolment trends, pass rate patterns and general quality of education over the 2018–2019 study period. It assessed local and stakeholders’ initiatives towards building a disaster-resilient education system. A mixed-methods approach integrated both quantitative and qualitative techniques to obtain in-depth narratives of lived experiences and quantitative aspects. A chi-square test provided sufficient ground to conclude that there were significant dropout patterns at a 95% confidence level. A paired *t*-test for the years 2018 (M = 64.59; SD = 12.61) and 2019 (M = 62; SD = 15.32) showed no significant difference (*t* [11] = 0.817, *p* = 0.432) at a 95% confidence interval. Concerted efforts are needed to avert the native pattern of school enrolment and academic results reflected in the study.

**Contribution:**

We therefore recommend that a holistic integrative disaster resilience framework between school, community and stakeholders showed great potential for the future.

## Introduction

As climate change–induced disasters continue to unfold, they pose a severe threat to the education system, particularly in already vulnerable communities (Briggs 2018; Conteh 2015). Local communities and other related stakeholders are expected to unite in building resiliency in education in pursuit of Sustainable Development Goal (SDG) 13.3. An evaluation of these efforts is critical to inform policy on disasters and development.

Disaster resilience is currently a critical and inevitable concept in the development discourse. According to Klein et al. (2003), the term resilience has its roots in ecology, where it is called *resiliere*, a Latin word implying the ability to bounce back or jump back. The United Nations Office for Disaster Risk Reduction (UNISDR) (2007) states that disaster resilience is the capacity of a system, community or society potentially exposed to hazards to adapt by resisting or changing to reach and maintain an acceptable level of functioning and structure. In this article, we adopt Agrawal’s (2018:2) definition that: ‘resilience is the ability of individuals, communities, organisations and states to adapt to and recover from hazards, shocks or stresses without compromising long-term prospects for development’.

Nearly 40 million children a year have their education interrupted by disasters (Watt 2019). Disasters may last a short period, but survivors can be involved with the disaster aftermath for months or even years (Chingombe & Musarandega 2021; Gibbs et al. 2019; Lazarus et al. 2003). Thus, the United Nations Educational, Scientific and Cultural Organization (UNESCO) (2017) underscores the need to provide education during and after a conflict or disaster to counter the negative effects of disaster disruption on the school system. Thus, schools can take a lead in building resilience and self-efficacy into their everyday culture and long-term planning towards stakeholder initiatives to build resiliency in education in pursuit of SDG 13.3 (Mutch 2014).

The conceptual underpinning of this article was influenced by the human capital development theory. The theory has it that knowledge, abilities and skills are regarded as invisible assets that are highly needed for sustainable development to occur (Wuttaphan 2020). McConnell et al. (2009:85) state that: ‘a more educated, better-trained person is capable of supplying a larger amount of useful productive effort than one with less education and training’. Therefore, educating the population helps to enhance human capital (McDermott 2012). A World Bank report asserts that education is one of the most powerful investments for reducing poverty and inequality and lays the foundation for sustained economic growth. Therefore, any threat to education is a menace to SDG4.

While education may rarely be a core focus in emergency response (Watt 2019), a resilient education system enables sustainable development by ensuring that human capital is built. Research on the role of schools in disaster preparedness, response and recovery is sparse, yet the school system is under threat from disasters (Conteh 2015; Mutch 2014; Striessnig, Lutz & Patt 2013; UNESCO 2018, 2019). Globally, climate change–related catastrophes gobble at least US$70–100 billion dollars annually in terms of adaptive capacity enhancement cost (Striessnig et al. 2013), putting the education system under severe threat. As for sub-Saharan Africa, the frequency and intensity of natural hazards are expected to increase because of global climate change. Accordingly, the region’s educational development initiatives are severely jeopardised, since a large proportion of the population is younger (Striessnig et al. 2013).

The focal area of this article is eastern Chimanimani, where the Cyclone Idai disaster disrupted many activities, including the education system (Chingombe & Musarandega 2021). The cyclone resulted in deaths and displacements, and other people moved away from the area altogether (Nhamo & Chikodzi 2021). The objectives of this article are to: (1) unveil the pre- and post-Cyclone Idai school enrolment patterns in selected schools in eastern Chimanimani, (2) analyse the impact of the disaster on school pass rate and (3) proffer options aimed at enlightening the school education system resilience in Chimanimani District. We strongly aver that the synergy of education and development concepts can possibly be brought to the fore by various policymakers.

## Methodology

### Description of the study area

**FIGURE 1:**
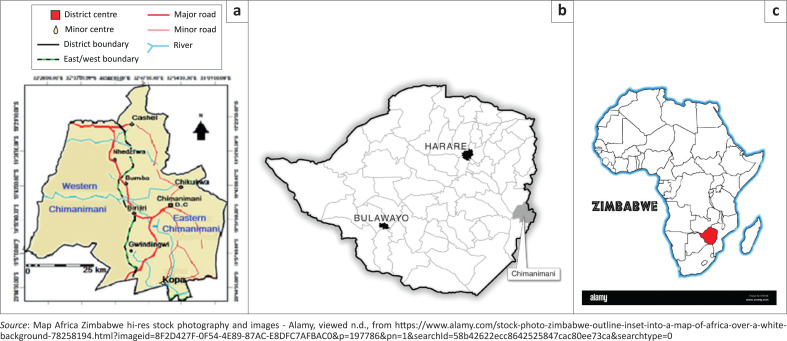
Maps showing the study area of: (a) Wester and Eastern Chimanimani District, (b) Chimanimani District within Zimbabwe and (c) Zimbabwe within the African continent.

Chimanimani East, which was mainly affected by the negative effects of Cyclone Idai, was selected for the study. The area lies in the Agro-ecological region 1 (Chingombe & Musarandega 2021). The region has average rainfall in excess of 1000 mm per year and droughts are a rare phenomenon. The major river in this area is Rusitu with its tributaries Mutsangadzi, Chipita, Haroni and Musapa near Chikukwa. The annual mean temperature is approximately 16 °C with the possibility of frost in winter (Chanza et al. 2020). The landscape is dominated by high and rugged terrain with an altitude of up to 6000 m above sea level. The vegetation is mainly savanna woodland to mountain grassland and broad-leaved evergreen forests. Figure 1 shows the locational details of the study area.

Chimanimani District has 134 940 people who are largely rural (95%) with a 95% literacy rate (ZIMSTATS 2013). About 1600 households, containing 9600 people, were directly affected by Cyclone Idai as stated by Copercious (2019) in Chanza et al. (2020). The livelihoods in the study area are basically centred on semisubsistence farming of horticulture produce (mainly potatoes, bananas, mangoes and sugarcane, among others), maize production and plantations of tea, coffee and timber (Chanza et al. 2020; Chingombe & Musarandega 2021).

In terms of infrastructure, the area has poor dust roads which become muddy and slippery in the rainy season, making it difficult to transport produce to markets (Musarandega et al. 2021). The foregoing physical and socio-economic conditions are underlain by vulnerabilities. The poor road network conditions easily cut these communities off in the event of heavy rains brought by cyclones. The Kopa area, which was the epicentre of destruction, lies at the confluence of the Rusitu, Chipita and Nyahode Rivers, making it highly prone to river flooding Chanza et al. (2020). In addition, eastern Chimanimani is situated close to the Indian Ocean, rendering the area vulnerable to cyclones because of the interplay of sea and ocean surface conditions.

### Methods

A mixed-methods approach embedded in a case study design was used to undertake the study. The use of case study and phenomenology gathers enriching information grounded in experience (Moustake 2009; Yin 2009), while the choice of using mixed research was premised on the belief that neither the qualitative nor the quantitative approach is superior, so we used both paradigms as complementary rather than conflicting entities (Creswell 2014; Tuckman 1999). The methodological pluralism or inclusiveness of eclecticism of the mixed methods results in superior outcomes compared to monomethod (Johnson & Onwuegbuzie 2004; Sieber 1973).

We selected eastern Chimanimani for our study, since it is home to information-rich respondents about the Cyclone Idai disaster. These include teachers, learners, parents, community leaders, nongovernmental organisations (NGOs), civic organisations and various government departments. Likewise, community households were randomly picked in the Rusitu area. A phenomenological research approach was thus desirable, hence the harnessing of in-depth interviews with snowballed participants, beginning with those who outspokenly participated in public meetings.

The relevant variables of interest were student enrolment, dropout rates and quality of education (related to access to sound infrastructure, qualified teachers, teaching and learning materials and pupils’ performance). The independent variable is the disruptive Cyclone Idai, in this case. We used descriptive statistics to deduce average values and percentages, which helped to make various generalisations. In quantitative techniques, correlational analysis on enrolments, dropout rates and pass rates were of interest. This gave advocacy to the link between the cyclone disaster and the school system.

The statistical results obtained were tested using the chi-square test and the paired *t*-test for enrolment and pass rate before and after the disaster, respectively. The chi-square test on enrolment losses was run in order to confirm whether there was a significant difference in the dropout rate before and after the catastrophe. The analysis was guided by dual hypotheses. Firstly, we anticipated that there was no association between the enrolment status and the year reference (H0). Secondly, the enrolment differed according to the year reference (H1). Furthermore, a paired *t*-test was used to determine the impact of the disaster on school pass rate. We hypothesised that there was no pass rate difference in studied schools before and after the cyclone disaster (H0). Thirdly, the school pass rate differed following the cyclone disaster (H1). Literature on Cyclone Idai and many other disaster-affected settings was reviewed. The literature and field observations were triangulated with the interview results in order to produce a balanced interpretation and conclusion on the variables of interest. On the other hand, qualitative data presentation was in the form of content quotes followed by their interpretation and narration of transcriptions and summaries.

### Ethical considerations

Permission to visit schools and collect data from schools was sought from the Chimanimani District Education Office. The study was undertaken during the coronavirus disease 2019 (COVID-19) period and all protocols were observed. The study participants joined out of their own willingness. No participant was forced to provide data. Participant’s names and those of schools involved were kept anonymous. Members were given the freedom to withdraw their participation at any stage of the study. Authors also sought consent from the study participants to cite the stated views as long as names were withheld.

## Findings

### Pre- and post-Cyclone Idai school enrolment patterns

The total enrolment for the 12 sampled schools before the cyclone was 9340 learners in March 2019. The total enrolment after the cyclone was 8103. Mean enrolment before the cyclone was thus 778, and after the cyclone, it was 675, reflecting a 13% decline in the sampled schools within a space of 2 months. Interviewed school authorities ascribed the decline in enrolment firstly to the death of some learners. Data obtained from the Chimanimani District Education Offices revealed that in the whole District, 104 learners were reported either as deceased or as missing. A total of 14 secondary school learners who lodged in the Kopa area alone were also swept away by floods, according to Ministry of Education documents availed to the researcher.

Furthermore, reduced enrolment resulted from the loss of some learners’ parents and guardians. According to the Chimanimani District Education Office data obtained, the cyclone left 71 learners orphaned. The majority of these learners were forced to eke out a living as vendors and domestic workers. A few privileged ones sought assistance from organisations, such as Higher Life Foundation. One dropout teenager traced from the list given by the District Education Office said that he has since resorted to illegal gold panning at Musanditeera Mountains and newly discovered gold fields at Bullock area in Ngorima area in Rusitu in order to survive. Researcher visits to Bullock and several other illegal mining sites helped to confirm the widely reported growing numbers of teenagers who have dropped out of school to engage in artisanal mining.

The school disruption also saw enrolment loss because of early marriages and unplanned pregnancies, as indicated by the reports from interviewed school authorities who work closely with disaster-affected communities. This tendency was common in the studied secondary schools. At one visited school, a headmaster noted seven girls who were married and ascribed this loss in education to over-exposure to sexual activities during displacements and in temporary shelter. St Charles Lwanga Secondary, the most hit school, had an enrolment loss of 61%. Therefore, the enrolment figures show that disasters are disruptive to the education system, as they lead to the death, dropout and forced transfer of learners from highly affected schools.

In Rusitu valley, most schools had their classroom blocks, teachers’ houses and ablution blocks seriously destroyed by winds and torrential rains. Ngangu Primary and Chimanimani Secondary schools experienced the same fate. Such devastation forced the schools to be temporarily closed as reconstruction was sought. One of the interviewed parents, who happens to have two children at Ndima Secondary School, alluded:

‘Most building structures and power lines in schools were destroyed. Water sources have been seriously affected in the Kopa community and schools. The loss of power means that water can no longer be pumped, posing a health hazard that compromises the welfare of learners and school staff for quite an extended period.’ (Participant 6, Parent, Ndima Secondary School, 12 October 2020)

Cyclone Idai also led to a disruption of staff establishment in some schools. The disaster caused the deaths of three teachers at Dzingire Primary School. Nyabamba Primary School also reportedly lost one teacher. Accordingly, the disaster caused loss of human resources and reduced quality of education in this disaster-prone part of the district, since many traumatised teachers continue to live and work in fear of yet another high-level disaster.

We computed a statistical record of student enrolment for selected schools over the 2018–2019 period. Heads of schools and the schools inspector provided questionnaire data on the enrolment trends across the study area. Table 1 reveals the findings from the interviews with heads and area education inspectors.

**TABLE 1:** Enrolment trends for sampled schools in Chimanimani.

Variable	School											
	1	2	3	4	5	6	7	8	9	10	11	12
2018 T1	877	1172	1156	583	712	757	681	263	859	889	733	694
2018 T3	870	1181	1130	578	710	760	678	257	855	897	736	686
Difference	−7	11	−26	−5	−2	+3	−3	+6	−4	+8	+5	−8
2019 T1	915	1197	1124	560	720	811	681	311	872	996	751	709
2019 T3	957	1178	839	203	700	780	568	99	809	746	605	619
Difference	+42	−19	−285	−357	−20	−31	−113	−212	−63	−170	−146	−90

Note: Numbers 1–12 represent the sampled schools.

T1, Term 1; T3, Term 3.

**FIGURE 2:**
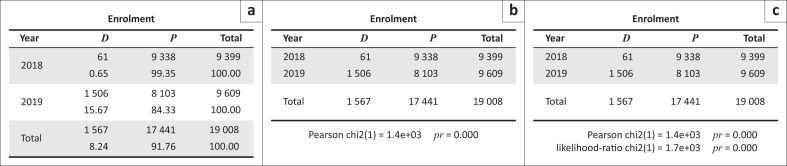
Statistical relationship of the enrolment changes before and after Cyclone Idai disaster in eastern Chimanimani.

The 2018 and 2019 figures reflect the pre- and post-catastrophe enrolment scenarios, respectively. A chi-square test was performed to assess the statistical significance of the enrolment changes before and after the disaster. Figure 2 shows the results obtained.

The results of the study indicated that there is a strong association between the enrolment position and the reference year. Put in another way, the proportion of dropouts in 2018 is significantly different from the year 2019 at 95% confidence level, since *p* < 0.05.

### Impact of the disaster on school pass rates

Using questionnaire data corroborated by interview assertions, we compiled Zimbabwe Schools Examination Council (ZIMSEC) examination pass rate data for the years 2018 and 2019, respectively. This was meant to help deduce pass rate trends before and after the Cyclone Idai disaster in order to link academic achievements and disasters. Table 2 gives details of the findings on the status of the pass rate.

**TABLE 2:** Pass rates before and after Cyclone Idai in selected schools in Chimanimani District.

Variable	School											
	1	2	3	4	5	6	7	8	9	10	11	12
Pre-Cyclone Idai pass rate (%)	65	86	88	52	63	71	64	58	70	57	53	48
Post-Cyclone Idai pass rate (%)	71	77	68	43	79	68	60	47	88	52	47	44

The mean pass rate before the cyclone in 2018 for the schools sampled was 64.6%. The mean pass rate after the cyclone in 2019 was 62%, giving a decline of 2.6% overall in the sample. Sampled schools 3 and 8 had huge declines of 20% and 11%, respectively.

**FIGURE 3:**
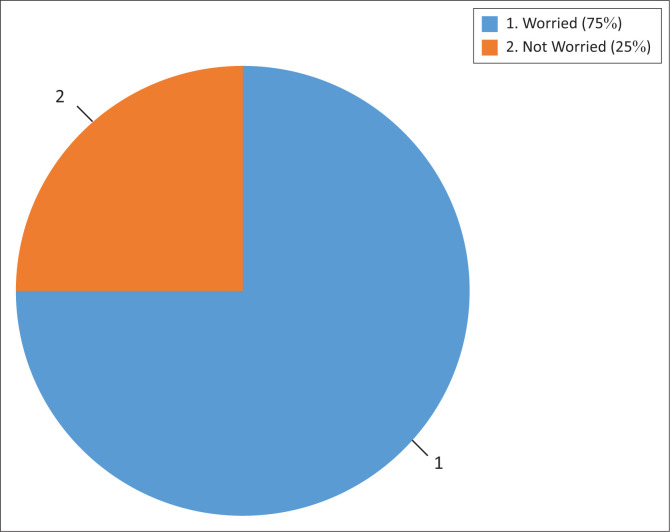
School heads’ perception of the decline in the school pass rate.

Going further, we undertook a paired *t*-test using the obtained data with a view to determine the significance of the pass rate before and after the cyclone disaster. The results of the conducted test are shown in Figure 3.

The computed paired *t*-test showed that there is no significant difference in terms of school pass rate for the years 2018 and 2019 (*p* = 0.432) at 95% confidence interval. In our study, we took the risk to commit a statistical error of failing to reject a null hypothesis when in fact, it should be rejected. The analysis results show that there is sufficient statistical evidence to conclude that the cyclone disaster led to lowered educational attainment, the outcome of which was not desired by the affected schools and the education system in general.

We interviewed 12 heads of the studied schools to ascertain whether they were worried about the slump in the pass rate. The results of their sentiments are reflected in Figure 3.

**FIGURE 4:**
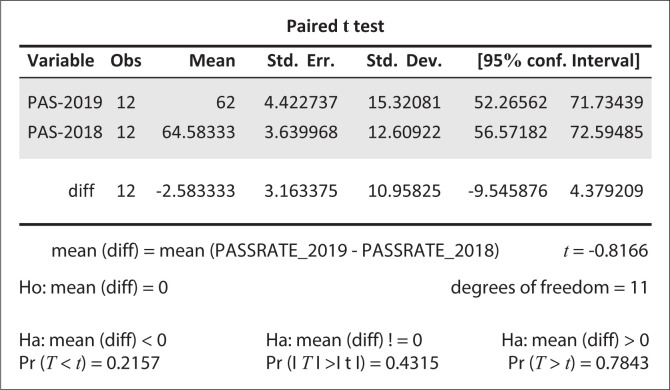
Summary of *t*-test results to compare the pass rate in selected schools before and after Cyclone Idai.

The outcome of the poll concurred with our conclusion on the *t*-test results (see Figure 4) that even a marginal decline is worrisome. A total of 9 (75%) out of 12 school heads expressed their worries about the decline in performance. The other three (25%) school heads indicated that they were not worried about the results. Our further analysis revealed that the two who failed to express concern with the results (schools 1, 5 and 9 in Table 2) were from boarding school setups, where the Cyclone Idai disaster did not cause serious devastation. We visited the three schools and confirmed that the teaching and learning infrastructure was intact. The laboratories were safe and learners were carrying out their experiments as usual. The disaster caused families from highly vulnerable settings to lose their shelter. As a post-disaster response strategy, learners from affected households were relocated to distant sites, where they could hardly reach school. Consequently, this added to the school dropout causes.

We triangulated our findings with narratives of other informants within seriously affected schools. The participants related the decline to excessive trauma, since such schools were hit hardest and had fatalities. Post-traumatic syndromes were high in learners who lost their peers. Psychosocial support programs stretched longer than the accelerated learning programs:

‘We had a huge decline in educational achievement because trauma in learners and staff was high after the death of colleagues, destruction of dormitories and protracted school closure. Even when schools opened there were protracted periods of psycho-social support programs affecting learning time.’ (Participant 4, School Senior Master, St Charles Lwanga Secondary School, 13 October 2020)

The majority of interviewed teachers also pointed out that a slight decline in pass rate impacts future enrolments heavily. The school authorities bemoaned excessive trauma to both staff and children. Some study participants, who also happen to be parents of some learners, expressed that they were more comfortable with upward deviations in pass rate as opposed to downturn scenarios typical of the post-Cyclone Idai one. When infrastructure was destroyed, there was also serious loss of educational materials. The loss of educational material translated to educational disturbance, particularly in the area of practical subjects, where learners benefit from constant use of teaching and learning equipment.

### Options for enlightening the school system resilience in Chimanimani District

**FIGURE 5:**
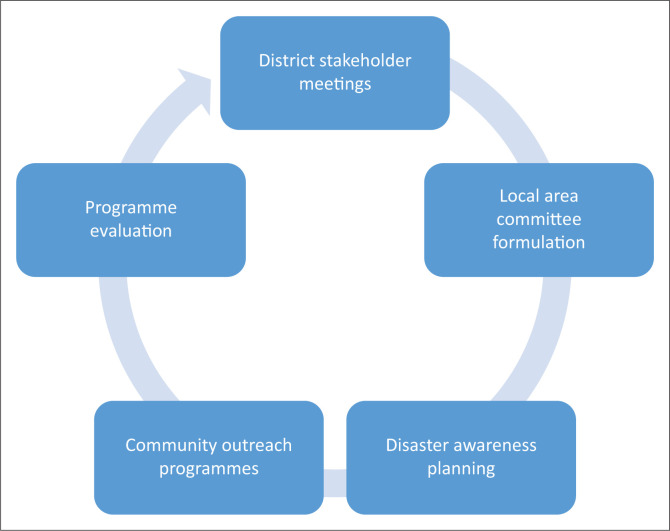
Local and stakeholder Disaster Risk Reduction (DRR) initiatives flow observed in Chimanimani District.

We traced the efforts on the ground by various stakeholders aimed at alleviating the Cyclone Idai disaster’s effects on the community and the education system in general. The insights from the various participants were thematically analysed by arranging them according to the nearness of connotation. The summarised flow of activities is depicted in Figure 5.

We used thematic content analysis in order to narrow down the stated list of initiatives depicted in Figure 5. Each time the participants raised a suggestion, we would synthesise its meaning and later compress the notes to narrow themes according to their nearness of connotation. Multistakeholder meetings were hailed, as they give room for diverse ideas to emerge. Local area committee formulation paved the way for practical participation of the local people in decision-making and disaster awareness planning. The majority of the members of the community preferred communities, because they represent their own interests in as far as disaster risk reduction is concerned. Participatory program evaluation was suggested with a view to confirm whether the undertaken community outreach packages were in line with set standards as well as the interests of the locals.

A total of 12 (100%) sampled schools showed evidence of disaster risk reduction work plans and psychosocial support programs being undertaken, courtesy of organisations such as World Vision, Terre des Hommes (TDH), Plan International, Regional Psychosocial Support Initiative, Towards Sustainable Use of Resources Organisation, United Nations Children’s Fund (UNICEF) and government. Other organisations just assisted during the emergency phase and went away, but the foregoing ones are still on the ground to assist the education sector to recover and build back better towards a resilient education system. The latter remained behind, since the long-term community engagement mandates extend deep into the post-disaster period.

We attended two workshops that were organised by each of the intervening agencies, namely the European Union, the International Institute of Rural Reconstruction and TDH. Through the initiatives, a wide spectrum of disaster management initiatives was taught to schools after Cyclone Idai under the theme ‘Empowering school communities for disaster preparedness and risk reduction through the formal education system’. Among other suggestions, school heads who attended the workshops widely called for the modernisation of the education sector, including a reduction in subscription charges. They stated that an improvement in information communication and technology systems, including cost reduction, helps a great deal in the post-disaster building of educational resilience, since learners will be in a position to use interactive platforms, such as WhatsApp, Facebook, YouTube and many others.

## Discussion

Disaster epics are often followed by a delayed but serious disaster impact on the educational performance of schoolchildren (Gibbs et al. 2019; Nguyen 2018). Although it is positive to find no statistically significant difference in those early years after the event, as in the Chimanimani case, the risk is that subsequent impacts on academic performance are overlooked, and thus without targeted interventions, children’s future academic trajectories are compromised. Nguyen et al. (2018) confirmed that exposure to disasters reduces the number of completed grades of children after they found a direct correlation between flood exposure and decreasing cognitive ability scores in Vietnam. Nhamo and Chikodzi (2021) pointed out that Cyclone Idai resulted in deaths and displacements which disrupted the normal operations in the area as people were forced to move away from the area. We therefore strongly aver in this article that early interventions are mandatory, so that no child is left behind by strict adherence to positivist quantitative conclusions.

Educational attainment and achievement are a function of uninterrupted learning time and the provision of adequate and relevant resources. Protracted disruption periods point to a bleak future for the education system (Kousky 2016; Nguyen 2018). The study results are congruent with the study of wildfires’ effects on academic performance in Australia by Gibbs et al. (2019), which showed that a disruptive event may not have apparent effects in the short term, but when considered over time, the effects are profound on child academic performance in the future. In addition, Watt’s (2019) study in Mozambique also showed that when children fail to attend school for an extended period because of a disaster, they face the danger of succumbing to child labour, early marriages and trafficking, as well as other risks. Many of them may not resume their education at all.

Disasters do seriously impact the stability and performance of learners. Cahill et al. (2011) noted a range of psychological and mental health issues associated with disaster shocks on children, that is, reduced sense of safety and security, self-worthy, social connection, self-efficacy and sense of purpose, hope and meaning. Psychosocial support programs are therefore mandatory to curb these effects. Children have greater trouble processing emotional trauma, causing post-traumatic stress disorder Kousky (2016). The effects can persist into adulthood and even the next generation (Heckman 2007).

The building of permanent school infrastructure is often considered a secondary priority, resulting in children being educated in temporary learning centres for years after a disaster event Watt 2019), a move that negatively affects the quality of the teaching and learning outcome. This resonates with Mavhura (2020), who equally avers the importance of having effective disaster preparedness rather than what is occurring in Zimbabwe, that is, remaining in the disaster–response–disaster cycle instead of a proactive preventive approach to cyclones. Such a development hinders progress towards education for all and SDGs, UNESCO (2000) avers.

The suggested means to improve the resilience of the education system in Chimanimani triangulate well with Thornley’s (2013) call for encouraging community-led organisation and action, understanding community complexity and diversity to develop and strengthen partnerships between communities and authorities. This bolsters community capital. To bolster community empowerment under the guidance of traditional leadership and cultural norms, values have been hailed earlier (Musarandega et al. 2018). As well, UNISDR (2015) notes that community capital envisages economic resources, assets, skills, information and knowledge, supportive network, access to services and shared community values. This is critical in the school system and community resilience.

## Conclusion and recommendations

Cyclone Idai caused widespread deaths and destruction of classroom blocks, furniture, bridges, power lines, water reticulation infrastructure, toilets and school income–generating projects. The disaster event increased schools’ vulnerability, reducing the quality of education in disaster-affected schools of eastern Chimanimani. The school enrolment and educational attainment were lowered. Negative coping mechanisms ranged from informal activities such as vending, illegal mining, giving into early marriages and at worst, indulging in commercial sex. Death also accounted for the attrition. The results of this study pave the way for future studies on the long-term effects of the Cyclone Idai disaster on the education system in the area.

The study therefore advances the following recommendations:

- Lived experiences must not be ignored in disaster management for schools’ resilience.
- Proactive action must be taken to curtail negative effects which are not very apparent in the short term but existing as accounted for by subjects interviewed.
- Psychosocial support initiatives are needed to curb the negative effects of disasters on the educational performance.
- Multistakeholder participation is highly encouraged in rebuilding the school system during the post-Cyclone Idai period and beyond.
